# Effect of oregano essential oil and benzoic acid supplementation to a low-protein diet on meat quality, fatty acid composition, and lipid stability of *longissimus thoracis* muscle in pigs

**DOI:** 10.1186/s12944-017-0535-1

**Published:** 2017-08-31

**Authors:** Chuanshang Cheng, Xiaming Zhang, Mao Xia, Zuhong Liu, Hongkui Wei, Zhao Deng, Chao Wang, Siwen Jiang, Jian Peng

**Affiliations:** 1 Department of Animal Nutrition and Feed Science, College of Animal Science and Technology, Huazhong Agricultural University, Wuhan, 430070 People’s Republic of China; 2 The Cooperative Innovation Center for Sustainable Pig Production, Wuhan, 430070 People’s Republic of China

**Keywords:** Low-protein diet, Oregano essential oil, Benzoic acid, Fatty acid composition, Lipid stability, Pigs

## Abstract

**Background:**

Consumers are becoming increasingly interested in food containing appropriately high concentration of intramuscular fat (IMF) and polyunsaturated fatty acids (PUFA). The supplementation of feed with antioxidants decreases degradation of lipids in muscles thereby enhances nutritional and sensory properties of meat. Two experiments were conducted to determine the effects of adding oregano essential oil (OEO) and benzoic acid (BA) to low-protein, amino acid-supplemented diets on meat quality, sensory profile, fatty acid composition, and lipid oxidation of *longissimus thoracis* (LT) muscle in pigs.

**Methods:**

In Exp. 1, 21 barrows were housed in metabolism cages and randomly allotted to 1 of 3 diets. The three diets were normal protein diet (NPD), medium protein diet (MPD) and low protein diet (LPD) with 1% and 2% less than NPD, respectively. In Exp. 2, 36 barrows were randomly divided into three experimental groups, namely, NPD, LPD, and identical LPD supplemented with blends of OEO (250 mg/kg feed) and BA (1000 mg/kg feed) (LPOB) groups.

**Results:**

No significant effects of diets on meat quality were observed in Exp. 1. The b*_45min_, tenderness, and IMF content in LPD muscle were higher than those in NPD and LPOB muscle. The LT muscle in LPD group contained a higher percentage of oleic acid (C18:1n-9) and a lower percentage of linoleic acid (C18:2n-6) than those in NPD group. Dietary LPOB improved oxidative stability, total superoxide dismutase, and glutathione peroxidase but decreased drip loss in LT muscle.

**Conclusions:**

These findings suggest that growing-finishing pigs fed with a low-protein, amino acid-supplemented diet show a high content of intramuscular fat in the *longissimus thoracis* muscle. Dietary LPOB enhances the anti-oxidative status by improving antioxidative capacity but deteriorates the sensory attributes by decreasing IMF content of meat.

**Electronic supplementary material:**

The online version of this article (doi:10.1186/s12944-017-0535-1) contains supplementary material, which is available to authorized users.

## Background

During the last few decades, the pork market has been subjected to several changes influenced by consumer demands, which have focused the production toward healthier, safer, and tastier meat. The intramuscular fat (IMF) is crucial for meat palatability, such as tenderness and juiciness, and is an important economic trait in pork production [1–3]. Besides the IMF amounts, consumers are becoming increasingly interested in food containing high concentration of polyunsaturated fatty acids (PUFA). PUFA are considered as functional ingredients to prevent cardiovascular disease [4, 5]. Hence, meat producers require producing and supplying meat that contains appropriately higher IMF and PUFA.

Dietary methods to manipulate the fat quantity and composition of pork have been previously reported, including low-protein or lysine-deficient diets [3, 6, 7]. A reduced protein diet promoted pork with higher IMF percentage which was more monounsaturated and less polyunsaturated even when balanced with essential amino acids [2, 8]. The tissue-specific increase of expression of stearoyl-CoA desaturase could be one reason for higher de novo synthesis of fatty acids in muscle of pigs fed reduced protein diets [9]. However, unsaturated fatty acids are more sensitive to oxidation than saturated fatty acids (SFA) and particularly vulnerable to peroxidative attack [10]. Moreover, the lipid oxidation in muscle is a major deterioration of nutritional and sensory properties of meat and meat products [11].

Synthetic and natural antioxidants have been used to delay the lipid oxidation in pork [12]. Herbal feed additives and organic acid have been suggested as desirable alternatives to benefit growth performance of the pigs by improving the gut health. Oregano essential oil (OEO) contains the natural and volatile aromatic compounds that exert different biological actions, such as antimicrobial, anti-inflammatory, and antioxidative activities [13]. Carvacrol and thymol, the two main phenolic derivatives that constitute about 81.9 and 3.5% of OEO, are principally responsible for the activities [13, 14]. In addition, other minor constituents such as γ-terpinene and ρ-cymene, two monoterpene hydrocarbons that constitute about 4.49 and 3.07% of OEO, respectively, also contribute to the activities [15, 16]. In our previous studies, we found that OEO can improve growth performance [17], intestinal barrier integrity [18], and antioxidant enzyme activities in the intestinal tissues [19].

Benzoic acid (BA), as a kind of organic acid, was authorized to be used in growing pigs at the dose of 0.5% to 1.0% by European Union in 2003 [20]. Certain research has proposed that BA can improve the growth performance [21], intestinal antioxidant capacity [22], and microecological balance [23]. In addition, previous studies have reported that the combination of herbal essential oils with organic acid produced a synergistic effect on antibacterial activity [24, 25]. However, there is little available information on the effects of long-term the combination of OEO and BA supplementation to a low-protein, amino acid-supplemented diet on antioxidant properties and fatty acid composition of pork production.

Therefore, we firstly evaluated the effects on meat quality of reducing the dietary protein concentration by one to three percentage units. Secondly, our objective was to investigate the effects of long-term OEO and BA supplementation to a reduced-protein, amino acid-supplemented diet on physical and chemical parameters, fatty acid composition, lipid stability, and sensory profile of *longissimus thoracis* (LT) muscle in growing-finishing pigs.

## Methods

### Animals, diets, and sampling

#### Experiment 1

In total, 21 healthy crossbred barrows (Landrace × Large White, 50 days old) with initial BW of 16.31 ± 1.26 kg were used in a 95-d trial. Pigs were placed in metabolism cages that were equipped with a feeder and a nipple drinker, fully slatted floors, and a screen floor. The pigs were randomly allotted to one of three diets based on BW in a randomized complete block design with seven replicate pigs per diet. The diets based on corn, distillers dried grains with solubles (DDGS), and soybean meal. Each diet supplemented with 150 mg aureomycin per kg feed as antibiotic growth promoter. A normal protein diet (NPD) with 170 and 156 g CP/kg with one percentage unit below the nutrient requirements of the National Research Council [26] for the growing and finishing period, respectively. A medium CP level diet (MPD) with 10 g CP/kg less than the NPD for the growing and finishing period, and a low level diet (LPD) with 20 g CP/kg less than the NPD for the two phases. All diets were fortified with Lys, Met, Thr and Trp to meet the requirements of growing-finishing pigs [27]. Nutrient contents of the experimental diets were presented in Table 1. All pigs were allowed ad libitum access to feed and water throughout the experiment.

**Table 1:** Nutrient analysis of the experimental diets (%, as-fed basis)^a^

	Growing pigs			Finishing pigs		
	NP	MP	LP	NP	MP	LP
Calculated analysis nutrients						
Net energy (Kcal/kg)	2475	2475	2475	2475	2475	2475
Analysed nutrients						
Crude protein	17.01	16.02	15.02	15.62	14.61	13.61
Dry matter	87.20	87.37	87.40	88.14	88.25	88.43
Total ash	6.10	6.16	6.13	5.82	5.87	5.93
Ether extract	4.10	4.07	4.08	4.13	4.12	4.12
Standardized ileal digestible amino acids						
Lysine	0.98	0.97	0.97	0.81	0.84	0.82
Methionine	0.28	0.27	0.28	0.22	0.22	0.21
Threonine	0.59	0.57	0.58	0.50	0.49	0.51
Tryptophan	0.17	0.16	0.17	0.14	0.15	0.13
Fatty acids (% of total fatty acids)						
C16:0	21.00	21.05	21.12	21.20	21.26	21.32
C18:0	3.85	3.79	3.78	3.68	3.56	3.45
C18:1n-9	19.40	19.50	19.70	19.64	19.75	19.92
C18:2n-6	49.90	49.60	49.42	49.50	49.42	49.36
C18:3n-3	1.41	1.39	1.40	1.41	1.40	1.39

^a^ *NP* normal protein, *MP* medium protein, *LP* low protein

On day 95, five barrows per treatments were slaughtered at a live weight of 98.95 ± 4.07 kg. Pigs were electrically stunned, exsanguinated, dehaired, and split down the midline according to standard commercial procedures. Then, fresh LT samples anterior to the 13th rib from the left side carcass were collected and used for the physical analysis.

#### Experiment 2

A total of 36 barrows (Large White × Landrace) with an initial body weight (BW) of 29.55 ± 1.27 kg were randomly allotted to 1 of 3 treatments with twelve replicates of one pig per replicate. Pigs were penned individually. The pig pens were kept in an environmentally controlled building with a temperature between 15 and 25 °C. All pigs were provided with ad libitum access to feed and water via semi-automatic individual feeders and nipple drinkers. Pigs in group I and group II were fed with the same NPD and LPD in Exp. 1, respectively, and group III received the same LPD supplemented with 250 mg of OEO and 1000 mg BA per kg of feed to substitute for aureomycin (LPOB). The OEO was provided by Meritech Bioengineering Company (Guangzhou, China). The components of OEO are shown in Additional file 1. The BA was purchased from Novus International (China).

On day 98, six barrows per treatments were slaughtered at a live weight of 106.53 ± 3.55 kg. Fresh LT samples anterior to the 13th rib from the left side carcass were collected and used for the physical analysis. Meanwhile, the LT muscle was removed from the left side carcass at the last lumbar vertebra and frozen at −20 °C for 3 days until chemical composition, oxidative stability, sensory evaluation, and fatty acid composition analyses were carried out.

### Physical parameters

All the physical parameters measurements were performed on 5 samples/group and 6 samples/group in Exp. 1 and Exp. 2, respectively. Marbling were scored using the National Pork Producer Council standards (marbling from 1 = devoid to 10 = abundant) [28]. Measurements of pH at 45 min and 24 h postmortem were performed on LT muscles using a pH meter (pH-STAR, SFK-Technology, Denmark). Meat color (MC) at 45 min and 24 h in Exp. 1 were measured for 3 times per sample on the cut surface using the OPTO-STAR meat color determinator (Matthäus). Color measurements (L*, a*, b*) in Exp. 2 were performed at 45 min postmortem from a mean of four random readings made with a chromameter (CR-300, Minolta Camera, Osaka, Japan), previously calibrated against a white tile according to the manufacturer’s manual. Drip loss and Cooking loss were determined by the method described by Honikel [29]. For drip loss determination, fresh meat samples (approximate 40 g) were held in a plastic box on a grid parallel to the fibre direction. The weight loss percentages after 1 (Exp. 1), 2, 4, and 8 days of storage at 4 °C were calculated. For cooking loss determination, a fresh slice from each sample was weighed (approximate 145 g), placed in a plastic bag, and cooked to an internal temperature of 70 °C in a 75 °C water bath. The internal temperature was monitored during cooking with a handheld temperature probe. The cooked samples were cooled for 30 min, blotted dry, and weighed. Cooking loss was expressed as the weight change percentage at the end of cooking. Warner-Bratzler shear force (WBSF) was determined in samples cooled at 4 °C for 24 h. Six cylindrical cores (10 mm × 10 mm) were removed from each sample parallel to the fiber direction, then, were sheared with a WBSF device attached to an Instron Universal Testing Machine (model 1011, Instron instrument, USA) with a 50-kg tension using a crosshead speed of 100 mm/min. The peak force (Newton/cm^2^) was recorded.

### Chemical parameters

Six samples/group of LT muscle were analyzed for dry matter, ash, and crude protein according to the methods of the AOAC [30]. Muscle IMF content was determined by Folch et al. [31]. The content of IMF was expressed as the weight percentage of wet muscle tissue. Each sample was analyzed in triplicate.

### Analyses of the fatty acid profile of IMF

Lipids from LT muscle samples were extracted in chloroform-methanol according to Folch et al. [31]. Fatty acid methyl esters were separated and determined with a CP-3800 Gas Chromatograph [32]. The fatty acids were identified by comparing the retention times of the peaks with those of known standards (Sigam Chemical Co., St. Louis, MO, USA). The response factors for the fatty acids were calculated using the same standard mixtures plus an internal standard [32]. Peaks were identified using standards where available (Sigma Chemical Co. Ltd., Poole).

### Measurement of antioxidant enzyme activity and oxidative stability

Approximately 800 mg of frozen muscle sample was weighed and homogenized on ice in 8 ml of 0.9% saline and then centrifuged at 2300×g for 10 min at 4 °C. The supernatant, which contained soluble enzymes and mitochondrial material, was used to measure enzyme activity and thiobarbituric acid reactive substances (TBARS) level in triplicate at appropriate dilutions. The activities of total superoxide dismutase (T-SOD), catalase (CAT), glutathione peroxidase (GPx), and total antioxidative capacity (T-AOC) in muscle were assayed using colorimetric methods with a spectrophotometer (Biomate 5, Thermo Electron Corporation, Rochester, NY, USA). The assays were conducted with commercial kits purchased from Nanjing Jiancheng Bioengineering Institute (Nanjing, Jiangsu, China) according to the manufacturer’s instructions. The total protein content of the supernatant was also determined by a commercial kit from Nanjing Jiancheng Bioengineering Institute (Nanjing, Jiangsu, China).

Oxidative stability of the LT samples were assessed by TBARS method at 1, 4, and 8 days of storage in a refrigerator at 4 °C. The analyses of TBARS were also conducted using commercial kits purchased from Nanjing Jiancheng Bioengineering Institute (Nanjing, Jiangsu, China) according to the manufacturer’s instructions. Determinations were carried out in triplicate. The content of TBARS, as an index of MDA, was expressed as nanomoles per milligram protein of muscle tissue.

### Acceptance test-eating quality

Eating quality characteristics were assessed in an acceptance test session according to AMSA [33]. Thirty persons, with previous experience, were recruited from the staff and students of the Department of Animal Science in the Faculty of Animal Nutrition and Feed Regulation. They were typical consumers (25–50 years old) of pork. A practicing session was performed before the test to allow consumers to become familiar with the test procedure, evaluation form, and sample presentation format. 

A total of 18 pork samples (6 animals per treatment) were evaluated, and each sample was evaluated five times. Meat pieces (2.5 cm thickness) of the three nutritional treatments were cooked, wrapped, cut, and kept warm prior to serving for acceptance test evaluation applying the method of Simitzis et al. [34]. Three attributes (flavor, juiciness, and tenderness) were evaluated with a ten-point descriptive linear continuous scale (1–10) with larger scores indicating a more favorable rating. Overall acceptance was rated on a nine-point hedonic scale (1–9) ranging from dislike extremely to like extremely. Each sample assessment involved three samples that represented the nutritional treatments (NPD, LPD, and LPOB). The paneling room was kept free of nonsample odors, artificial lighting was used, and the temperature was kept constant (approximately 20 °C).

### Statistical analysis

Each pig was considered as the experimental unit. The data were analyzed using the Statistical Analysis System (version 8.1; SAS Institute, Cary, NC, USA). Data for physical and chemical parameters, antioxidant enzyme activities, and fatty acid profile of IMF were analyzed by one-way ANOVA where the diet was the main effect. The data on drip loss and TBARS parameters during storage time in Exp. 2 were assessed by a two-way Analysis of Variance (ANOVA) with dietary treatment, time, and their interaction as effects. The attributes of eating quality (flavor, juiciness, tenderness, and overall acceptance) were analyzed using an ANOVA procedure, which contained the fixed effect of nutritional treatment and the effect of animal nested within nutritional treatment as a random effect, with the intention to correct the animal individual differences within treatment. The means were calculated using the least square method and presented with the standard error of the mean (SEM). When a significant *p*-value (*P* < 0.05 or *P* < 0.10) was observed in ANOVA, means of dietary treatments were compared using Duncan’s multiple comparison, whereas *P* < 0.10 was considered a tendency.

## Results

### Physical parameters

#### Exp 1

The meat quality characteristics of pigs are described in Table 2. There was no signifcant effect of treatment on pH values, meat color values, drip loss_24h_, and IMF content (*P* > 0.05).

**Table 2:** Effects of reduced-protein, amino acid-supplemented diets on meat quality of pigs

Item^a^	NPD	MPD	LPD	SEM^b^	*P*-value
pH, 45 min	6.17	6.26	6.22	0.07	0.880
pH, 24 h	5.63	5.61	5.68	0.03	0.929
MC (45 min)	70.90	69.94	66.30	1.09	0.198
MC (24 h)	51.69	49.55	48.79	1.03	0.528
Drip loss (24 h), %	0.95	1.07	0.96	0.04	0.568
Intramuscular fat, %	2.20	2.31	2.46	0.17	0.566

^a^ *MC* meat color, *NPD* normal protein diet, *MPD* medium protein diet, *LPD* low protein diet

^b^Standard error of mean, *n* = 5

#### Exp 2

**Fig. 1:**
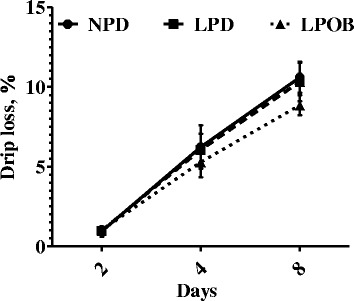
*Longissimus thoracis* muscle drip loss in relation to storage time at 4 °C. NPD = normal protein group; LPD = low protein, amino acid-supplemented group; LPOB = 250 mg/kg oregano essential oil and 1000 mg/kg benzoic acid group. Effects of treatment, *P* < 0.01; time, *P* < 0.001; time ∗ treatment, *P* = 0.139

Compared with the LPD group, the NPD and LPOB group showed a lower (*P* < 0.05) b*_45min_. There was a tendency to decrease (*P* = 0.078) marbling score of LT muscle in pigs fed LPOB diet compared to dietary LPD. No effects (*P* > 0.05) of dietary treatments were detected on pH values, L*_45min_, a*_45min_, cooking loss, and shear force (Table 3). The changes of drip loss of pork LT muscle in relation to dietary treatment (NPD, LPD, and LPOB) and storage time at 4 °C are shown in Fig. 1. Drip loss was significantly affected by storage time (*P* < 0.01) and dietary treatment (*P* < 0.001). No interaction between time and treatment was observed (*P* > 0.05).

**Table 3:** Effects of dietary treatments on physical parameters of *longissimus thoracis* muscle in pigs

Item^a^	NPD	LPD	LPOB	SEM^b^	*P*-value
pH, 45 min	6.34	6.51	6.55	0.04	0.324
pH, 24 h	5.66	5.73	5.78	0.03	0.248
Marbling score	2.00^ab^	2.20^a^	1.67^b^	0.10	0.078
Color indices, 45 min					
Lightness, L	39.89	37.39	37.42	0.68	0.236
Redness, a	4.58	5.04	4.41	0.29	0.680
Yellowness, b	10.47^b^	11.03^a^	10.34^b^	0.14	0.025
Cooking loss, %	25.28	24.36	23.51	0.55	0.463
Shear force, N/cm^2^	44.13	45.26	46.94	1.67	0.801

Means within a row with different superscript letters are significantly different (*P* < 0.05) or tendency (*P* < 0.1)

^a^ *NPD* normal protein group, *LPD* low protein, amino acid-supplemented group, *LPOB* 250 mg/kg oregano essential oil and 1000 mg/kg benzoic acid group

^b^Standard error of mean, *n* = 6

### Chemical parameters

Pigs fed with LPD had greater (*P* = 0.054) IMF in LT muscle compared with pigs fed with the NPD or LPOB diet (Table 4). Dietary treatments did not affect muscle moisture, crude protein and ash content (*P* > 0.05).

**Table 4:** Effects of dietary treatments on chemical parameters of *longissimus thoracis* muscle in pigs

Item^a^	NPD	LPD	LPOB	SEM^b^	*P*-value
Moisture, %	74.33	74.53	74.90	0.10	0.114
Crude protein, %	22.44	22.08	22.03	0.12	0.359
Ash, %	2.05	1.80	1.77	0.08	0.297
Intramuscular fat, %	1.94^b^	2.21^a^	1.94^b^	0.05	0.054

Means within a row with different superscript letters are significantly different (*P* < 0.05) or tendency (*P* < 0.1)

^a^ *NPD* normal protein group, *LPD* low protein, amino acid-supplemented group, *LPOB* 250 mg/kg oregano essential oil and 1000 mg/kg benzoic acid group

^b^Standard error of mean, *n* = 6

### Sensory profile

The values for tenderness, juiciness, flavor, and overall acceptance of LT muscle sensory profile are reported in Table 5. A LPD diet significantly improved (*P* < 0.05) tenderness of LT muscle compared with the NPD and LPOB diet. No significant difference in the ratings of juiciness, flavor, and overall acceptance of pork LT was detected among nutritional treatment groups (Table 5).

**Table 5:** Effects of dietary treatments on sensory parameters of pork eating quality in pigs

Item^a^	NPD	LPD	LPOB	SEM^b^	*P*-value
Tenderness	3.83^b^	4.28^a^	3.87^b^	0.06	<0.01
Juiciness	4.84	4.97	4.89	0.06	0.646
Flavour	4.69	4.63	4.44	0.05	0.165
Overall acceptance	5.26	5.42	5.19	0.07	0.598

Means within a row with different superscript letters are significantly different (*P* < 0.05)

^a^ *NPD* normal protein group, *LPD* low protein, amino acid-supplemented group, *LPOB* 250 mg/kg oregano essential oil and 1000 mg/kg benzoic acid group

^b^Standard error of mean, *n* = 6

### Fatty acid profile of IMF

The fatty acid profile of total fat in LT muscle is shown in Table 6. Compared with the NPD group, the LPD group contained a higher (*P* < 0.05) percentage of oleic acid (C18:1n-9) and monounsaturated fatty acids (MUFA) and a lower percentage of C18:2n-6 (*P* < 0.05) and PUFA (*P* = 0.089). No significant differences (*P* > 0.05) in fatty acid composition was detected between NPD and LPOB.

**Table 6:** Effects of dietary treatments on fatty acid composition (%) of *longissimus thoracis* muscle

Item^a^	NPD	LPD	LPOB	SEM^b^	*P*-value
C14:0	1.16	1.01	1.33	0.07	0.141
C16:0	23.52	23.07	24.39	0.54	0.620
C16:1n-9	3.06	2.97	3.09	0.14	0.945
C17:0	0.19	0.20	0.22	0.01	0.494
C18:0	11.63	12.82	12.02	0.30	0.279
C18:1n-9	38.02^b^	40.20^a^	39.56^ab^	0.36	0.029
C18:2n-6	12.69^a^	10.10^b^	11.06^ab^	0.41	0.015
C18:3n-3	0.29	0.25	0.24	0.01	0.230
C20:0	0.15	0.15	0.16	<0.01	0.851
c20:2n-6	0.32	0.29	0.30	0.01	0.421
c20:3n-3	0.27	0.30	0.29	0.01	0.630
C20:4n-6	2.30	2.39	2.48	0.14	0.875
C22:5n-3	0.51	0.53	0.50	0.04	0.951
SFA^c^	36.65	37.25	38.12	0.46	0.452
MUFA^d^	41.08^b^	43.17^a^	42.64^ab^	0.36	0.039
PUFA^e^	16.38^a^	13.86^b^	14.87^ab^	0.48	0.089

The fatty acid results were presented as g/100 g fatty acids (wt%). Means within a row with different superscript letters are significantly different (*P* < 0.05) or tendency (*P* < 0.1)

^a^ *NPD* normal protein group, *LPD* low protein, amino acid-supplemented group, *LPOB* 250 mg/kg oregano essential oil and 1000 mg/kg benzoic acid group

^b^Standard error of mean, *n* = 6

^c^Saturated fatty acids (SFA) percentage is the sum of 14:0, 16:0, 17:0, 18:0, and 20:0

^d^Monounsaturated fatty acids (MUFA) percentage was calculated as the sum of c16:1n-9 and c18:1n-9

^e^Polyunsaturated fatty acids (PUFA) percentage was calculated as the sum of 18:2n-6, 18:3n-3, 20:2n-6, 20:3n-3, 20:4n-6, and 22:5n-3

### Oxidative stability and antioxidant enzyme activities

**Fig. 2:**
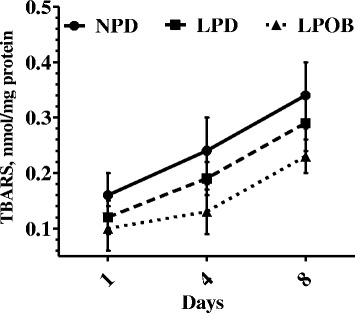
Oxidative stability during refrigerated storage at 4 °C of *Longissimus thoracis* muscle. NPD = normal protein group; LPD = low protein, amino acid-supplemented group; LPOB = 250 mg/kg oregano essential oil and 1000 mg/kg benzoic acid group; TBARS, thiobarbituric acid reactive substances. Effects of treatment, *P* < 0.001; time, *P* < 0.001; time ∗ treatment, *P* = 0.309

The dietary inclusion of OEO and BA decreased (*P* < 0.001) lipid peroxidation in LT muscle compared with the NPD and LPD diet. Times of storage also affected oxidative stability in LT muscle (*P* < 0.001) (Fig. 2). No interactions between time and treatment were detected (*P* > 0.05).

Antioxidative enzyme activities in LT muscle are shown in Table 7. Pigs fed with LPOB improved (*P* < 0.05) T-SOD, GSH-Px, and T-AOC activities compared with pigs fed with the NPD and LPD diet. No main effects (*P* > 0.05) were observed in CAT activities of LD muscle among three groups.

**Table 7:** Effect of dietary treatments on *Longissimus thoracis* antioxidative enzyme activities of growing-finishing pigs

Item^a^	NPD	LPD	LPOB	SEM	*P*-value
T-SOD (U/mg prot)	24.72^b^	22.77^b^	29.77^a^	0.94	<0.01
CAT (U/mg prot)	0.78	0.37	0.52	0.11	0.285
GSH-Px (U/mg prot)	3.32^b^	3.12^b^	7.29^a^	0.62	<0.01
T-AOC (U/mg prot)	0.50^b^	0.48^b^	0.60^a^	0.02	0.011

Means within a row with different superscript letters are significantly different (*P* < 0.05)

^a^ *NPD* normal protein group, *LPD* low protein, amino acid-supplemented group, *LPOB* 250 mg/kg oregano essential oil and 1000 mg/kg benzoic acid group, *T-AOC* total antioxidative capacity, *T-SOD* total superoxide dismutase, *CAT* catalase, *GPx* glutathione peroxidase

^b^Standard error of mean, *n* = 6

## Discussion

Higher IMF content has potential sensory benefits, such as deposition of fat within the muscle could enhance meat quality, i.e., tenderness and flavor [35, 36]. Different feeding strategies have been actively used in meat production to improve IMF [7]. Animal nutrition has a major impact on fat quantity and composition, as seen with low protein or lysine deficient diets that dramatically increase the level of IMF [2, 3, 6]. In the present study, the results of Exp. 1 indicated that pigs can feed low protein diet reducing dietary protein concentration by one to three percentage units without affecting meat quality of pigs. However, numerically, the values of IMF increased 11.8% in the LPD group compared with the NPD group, indeed, the results of Exp. 2 demonstrated that the IMF level in LT muscle of pigs was increased by LPD compared with NPD. In some cases, the effect has been explained by the different slaughter age [37] and slaughter weight [38] between the two trials, which were greater in Exp. 2 than in Exp. 1. Normal growth conditions, fat deposition of pigs occurs mainly in the late stages of fattening [39]. Therefore, the effect of dietary treatment on IMF was reflected in Exp. 2. The same result was observed in pigs fed with reduced protein diets balanced with five amino acids from 40 kg to 115 kg live weight [3]. On the other hand, meat from NPD and NPOB groups with high IMF content exhibited high b* values. Similarly, Alonso et al. [3] reported that low-protein diets increased the yellowness values of LT muscle. Sensory profile results showed that the muscle steaks with the higher IMF content, from regime LPD, had the higher scores for tenderness which agree with the findings of other researchers [2, 3] who reported that feeding protein-deficient diets improved the tenderness. There is no previously published research concerning the effects of the administration of a combination of OEO and BA on IMF content in muscles. Noteworthy, blends of OEO and BA substitute for antibiotic in a reduced-crude protein diet can reduce IMF, marbling scores, b*_45min_ value and tenderness of LT muscle in the present study. Some studies have suggested that the addition of OEO alone to the diet didn’t affect IMF content of animals [34, 40]. Kelly et al. [41] suggested that the average back fat thickness of the males was significantly (5%) increased as a result of aureomycin supplementation. Cho et al. [42] used mice to demonstrate that subtherapeutic antibiotic treatment promotes adiposity. Therefore, the use of blends of OEO and BA instead of antibiotics may affect lipid metabolism in LT muscle.

In addition to the quantity of lipids, The crude protein restriction during growing-finishing period resulted in an increase of MUFA and oleic acid (C18:1n-9) and in a decrease of linoleic acid (C18:2n-6) in accordance with Wood et al. [3] C18:1n-9 is the main product of de novo fat synthesis in the pig, and its concentration increases as the pig contains more IMF content. Wood et al. [3] also reported that a low-protein, amino acid-supplemented diet increased the percentages of C18:1n-9 and decreased the level of of C18:2n-6. No data regarding fatty acid composition of pork derived from animals fed with a combination of OEO and BA can be found in the literature, and data regarding OEO alone are scarce [43].

Lipid peroxidation is a natural phenomenon that has a significant effect on meat quality [44]. This study is the first to demonstrate that dietary supplementation with a combination of OEO and BA in growing-finishing pigs can beneficially increase T-SOD, GPx, and T-AOC activities in muscle which indicate an improved antioxidative capacity. These findings were in accordance with our previous studies reporting the positive effect of OEO on the activities of antioxidant enzymes in vitro and in vivo [17, 19]. Concerning the possible effects of the BA, the dietary inclusion of 0.5% BA increased GSH-Px activities in the liver of piglets [45]. High antioxidative enzymes may protect the lipid from oxidative damage [46]. The protection against lipid oxidation in the LPOB group was also confirmed by lower TBARS concentration and drip loss in the LPOB group during refrigerated storage at 4 °C. The protective effect of OEO, when used as a dietary supplement, on meat lipid oxidation has already been reported [17, 34, 40]. In contrast, Simitzis et al. [34] did not find any effect on stored pig meat when different concentrations of oregano essential oils (0.25, 0.5 and 1 mL/kg feed) were added to the diet. Furthermore, the key to minimizing drip loss was to protect the integrity of cell membranes from freeze injury, which increases their permeability and results in leakage of sarcoplasmic fluid [47]. Dietary supplementation with OEO and BA may preserve the fluidity of the membranes, which could otherwise be adversely affected by oxidative changes in the phospholipids [48]. These results agree with previously published reports, in which to the drip loss appeared to in pigs fed with diets supplemented with OEO [17, 43]. Therefore, results indicated that the LPOB diet improved the antioxidative status in LT muscle, extending the shelf life of meat.

## Conclusions

In this study, growing-finishing pigs fed with a reduced-protein, amino acid-supplemented diet showed a higher content of intramuscular fat in the *longissimus thoracis* muscle and a changed fatty acid composition towards a higher percentage of oleic acid and a lower percentage of linoleic acid in *longissimus thoracis* muscle. Low protein diets supplemented with the combination of oregano essential oil and benzoic acid may be an effective means to increase the growing-finishing pigs antioxidant status, to prevent lipid oxidation and, thus, to delay meat shelf-life. However, the long term supplementation blends of oregano essential oil and benzoic acid to low protein diet will reduce the content of intramuscular fat in LT muscle, which have a poor sensory characteristic.

## Additional file

Additional file 1: Chemical composition of oregano essential oil. (PDF 256 kb)

## Abbreviations

BA: Benzoic acid
CAT: Catalase
DDGS: Distillers dried grains with solubles
GPx: Glutathione peroxidase
IMF: Intramuscular fat
LPD: Low protein diet
LPOB: Low protein diet supplemented with oregano essential oil and benzoic acid
LT: Longissimus thoracis
MC: Meat color
MPD: Medium level diet
MUFA: Monounsaturated fatty acids
NPD: Normal protein diet
OEO: Oregano essential oil
PUFA: Polyunsaturated fatty acids
SFA: Saturated fatty acids
T-AOC: Total antioxidative capacity
TBARS: Thiobarbituric acid reactive substances
T-SOD: Total superoxide dismutase
WBSF: Warner-bratzler shear force
