# Supplementary material for: Effect of oregano essential oil and benzoic acid supplementation to a low-protein diet on meat quality, fatty acid composition, and lipid stability of longissimus thoracis muscle in pigs
Source: Lipids Health Dis. 2017 Aug 31;16:164. doi: 10.1186/s12944-017-0535-1 (PMC5577677; doi:10.1186/s12944-017-0535-1)
Supplement: Additional file 1: — Chemical composition of oregano essential oil. (PDF 256 kb) [file 12944_2017_535_MOESM1_ESM.pdf]

Additional file 1 Chemical composition of oregano essential oil

Components [a]	Composition %
α-Thujene/α-Pinene	0.66
Camphene	0.09
β-Pinene	0.07
Sabinene	0.04
Myrcene	0.86
α-Phellandrene	0.08
α-Terpinene	0.58
Limonene	0.13
1,8-Cineole+β-phellandrene	0.09
β-Ocimene	0.07
γ-Terpinene	4.49
3-Octanone	0.07
ρ-Cymene	3.07
Terpinolene	0.04
3-Octanel	0.01
1-Octen-3-ol	0.24
Dimethyl styrene	0.01
Trans-Sabinene hydrate	0.10
Linalool	0.28
Cis-Sabinene hydrate	0.06
1-Terpilool	0.04
Terpine-4-ol	0.34
Carvacrol methyl ether	0.22
β-Caryophyllene	1.41
Dihydrocarvone	0.08
α-Humulene	0.14
α-Terpineol	0.16
Bomeol	0.30
β-Bisaholene	0.70
Caryophyllene oxide	0.14
Thymol	3.50
Carvacrol	81.92
Total	99.99

[a] The data were provided by Meriden Animal Health Ltd.
